# Carbon Nanotube Field Emitters Synthesized on Metal Alloy Substrate by PECVD for Customized Compact Field Emission Devices to Be Used in X-Ray Source Applications

**DOI:** 10.3390/nano8060378

**Published:** 2018-05-29

**Authors:** Sangjun Park, Amar Prasad Gupta, Seung Jun Yeo, Jaeik Jung, Sang Hyun Paik, Mallory Mativenga, Seung Hoon Kim, Ji Hoon Shin, Jeung Sun Ahn, Jehwang Ryu

**Affiliations:** 1 Department of Physics, Kyung Hee University, Seoul 02453, Korea; sjpark1112@khu.ac.kr (S.P.); amargupta@khu.ac.kr (A.P.G.); sjyeo@khu.ac.kr (S.J.Y.); 2 CAT Beam Tech Co., Ltd., Seoul Biohub, 117-3, Hoegi-ro, Dongdaemun-gu, Seoul 02455, Korea; jijung@catbeamtech.com (J.J.); radpsh@gmail.com (S.H.P.); 3 Department of Radiology, Vinmec International Hospital, Ha Noi 10000, Vietnam; 4 Department of Information Display, Kyung Hee University, Seoul 02453, Korea; mallory@khu.ac.kr; 5 Department of Radiology, Asan Medical Center, University of Ulsan College of Medicine, Seoul 05505, Korea; kimsh6713@gmail.com (S.H.K.); jhshin@amc.seoul.kr (J.H.S.)

**Keywords:** carbon nanotubes, PECVD, metal alloy, field emission, compact field emission device, X-ray source

## Abstract

In this study, a simple, efficient, and economical process is reported for the direct synthesis of carbon nanotube (CNT) field emitters on metal alloy. Given that CNT field emitters can be customized with ease for compact and cold field emission devices, they are promising replacements for thermionic emitters in widely accessible X-ray source electron guns. High performance CNT emitter samples were prepared in optimized plasma conditions through the plasma-enhanced chemical vapor deposition (PECVD) process and subsequently characterized by using a scanning electron microscope, tunneling electron microscope, and Raman spectroscopy. For the cathode current, field emission (FE) characteristics with respective turn on (1 μA/cm^2^) and threshold (1 mA/cm^2^) field of 2.84 and 4.05 V/μm were obtained. For a field of 5.24 V/μm, maximum current density of 7 mA/cm^2^ was achieved and a field enhancement factor β of 2838 was calculated. In addition, the CNT emitters sustained a current density of 6.7 mA/cm^2^ for 420 min under a field of 5.2 V/μm, confirming good operational stability. Finally, an X-ray generated image of an integrated circuit was taken using the compact field emission device developed herein.

## 1. Introduction

Due to their strong C–C covalent bonds and high aspect ratio [1], carbon nanotubes (CNTs) exhibit extraordinary properties, such as high thermal conductivity, good chemical stability, and high mechanical strength. These properties make developing CNTs as cold field emitters very appealing for X-ray, microwave, and field emission display applications [2,3,4].

The CNT field emitter is commonly grown on a rigid semiconducting and insulating substrate, such as silicon or glass, to facilitate the formation of uniform nanoscale catalyst particles necessary for the assisted-growth of CNTs [5]. However, due to the relatively low electrical conductivity of the interface between the CNT emitter and aforementioned substrates, emitters grown in such manner have high turn on and unstable field emission [6]. Li et al. synthesized CNTs on a metal alloy substrate by coating its surface with Ni before synthesis. Given that catalyst seeds, such as Ni, Cr, Fe, Co, and Mo, are needed for the nucleation of CNTs, many reports emphasize the direct growth of CNTs on metal alloy substrates such as alloys of Ni, Fe, and the Cr (NFC alloy) system [7,8,9,10,11]. By using these metal alloy substrates (with or without surface modification), separate processes involving the sputtering of the above mentioned active catalysts and/or reagents, such as HNO_3_ and HCl, can be avoided. This ultimately makes the process much simpler and economical. Moreover, epitaxial growth of CNTs on NFC alloy yields superior intrinsic qualities, such as high electrical conductivity and mechanical properties [7], making NFC alloy the best substrate choice for direct synthesis of CNTs. 

We have recently proposed a simpler and economical way to directly synthesize CNT emitters on a metal alloy substrate (type of alloy: Hitachi Metal Corp., Tokyo, Japan, YEF 426, Ni-42%, Fe-52%, Cr-6%) with sufficient field emission for a custom built, open type X-ray system that can be used for biomedical applications [12]. The application of cold cathode emitters in X-ray source systems has gained much attention because of digitalized active control switching and relatively lower radiation emission compared to traditional thermionic emitters [13]. Although Si nanorods [14], ZnO nanorods [15], Mo [16], MoS_2_ [17], diamond films [18], graphene [19] etc. could be used as cold cathode emitters, none of them have proven to be as efficient as CNT-based emitters. Recently, FDA has approved the commercialization of a CNT emitter-based X-ray machine, developed by O. Zhou et al. [20]. Irrespective of the types of the cold cathode emitter, the prime focus in the development of both sealed or open-type X-ray source systems is the assembly of the field emission device in the electron gun. A small and compact field emission device is crucial to the development of portable, cheap, and small-sized X-ray source systems. In addition, a compact field emission device will highly facilitate multi-X-ray source systems that can be employed in novel imaging systems such as computed tomography, 3D Breast Tomosynthesis, etc. [21]. Although different types of compact field emission devices have been reported [22], none of them are sustainable nor utilize the conventional electron gun which is widely available in the market.

Towards the development of simple and economical CNT field emitters for X-ray source applications, we have optimized the plasma condition inside the PECVD chamber to grow high performance CNT emitters on a metal alloy substrate, without any pre-processing, sputtering of catalyst nor reduction with reagents. The CNT emitters are custom developed for the assembly of a compact field emission device that can replace thermionic emitters in conventional electron guns, and can play a significant role in the development of multi X-ray source systems.

## 2. Materials and Methods 

**Figure 1:**
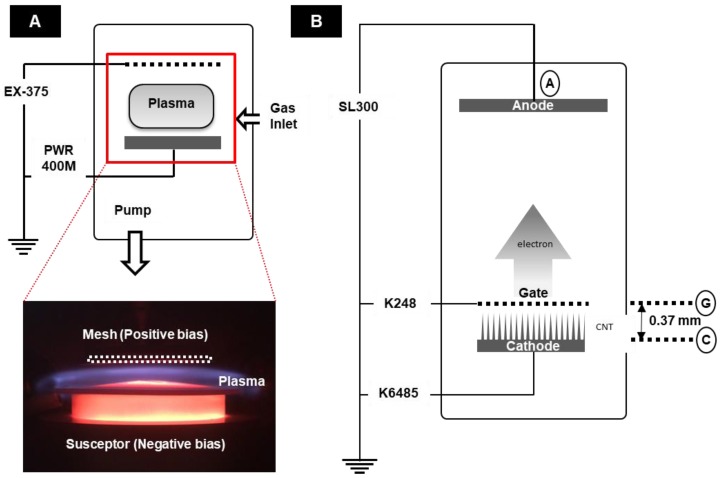
(**A**) Optical image and schematic diagram of plasma-enhanced chemical vapor deposition (PECVD); (**B**) Schematic diagram of field emission (FE).

Ellipsoid shape metal alloy substrates having thickness of 100 μm and emission area of 0.1377 cm^2^ with composition of Ni 42%, Cr 6%, and Fe 52%, were chosen for the synthesis of CNTs by a custom built diode PECVD process. Prior to growing, a basic cleaning procedure was performed in ultrasonic bath using acetone and followed by isopropyl alcohol (IPA). Each of the cleaning procedures took 10 min. After cleaning, the substrates were placed on a steel substrate holder, which was placed above a ceramic holder surrounding a graphite heater. The distance between two electrodes (substrate and mesh) was 10 mm, heater temperature was set at 900 °C, and base pressure of the chamber was maintained at 3.5 × 10^−6^ Torr. The graphite heater was heated at 20 °C/min. The anode voltage was supplied by EX-375 (Takasago LTD, Tokyo, Japan), whereas the cathode voltage was supplied by PWR400M (Kikusui Electronics Corp., Kanagawa, Japan). Growing procedures were performed in 3 steps, namely pre-treatment, growth, and post-treatment. During all these steps, the temperature of the reactor was maintained at 900 °C. In NH_3_ atmosphere, pre-treatment pressure was 4.2 Torr and the applied voltage between the two electrodes was 500 V. The 500 V DC is the optimum voltage required to form a plasma sheath right above the susceptor. The value was determined experimentally. The pre-treatment was carried out for 10 min and to form the nucleation sites for CNT growth on the surface of the metal substrates. Nucleation sites were formed due to etching of the substrate by NH_3_ plasma [23]. It has been reported before that the pre-treatment with NH_3_ plasma forms better nucleation sites compared to pre-treatment with NH_3_ gas [24]. Subsequently, NH_3_ and C_2_H_2_ were respectively inserted together to synthesize CNTs at 70 and 30 sccm for 30 min. During the growth process, pressure was maintained at 5 Torr. The post-treatment involved the annealing of CNTs under NH_3_ plasma for 1 min at 900 °C followed by natural cooling. The decreasing ratio of acetylene to ammonia during post-treatment helps to remove the unwanted amorphous carbon and nanoparticles on CNT. This improves the crystallinity of the graphitic surface and field emission characteristics [25,26]. Figure 1 shows both the optical image and schematic diagram of optimized plasma formation, during the PECVD growth process. We found that, in the PECVD process, plasma profile control is key to the proper synthesis of CNTs on metal substrates. Many studies have been reported on the optimization of the plasma profile through voltage bias, heater temperature, gas flow, and pressure [5,8]. In the present study, we optimized the plasma profile by controlling the anode voltage and pressure in a way that the plasma sheath is formed right above the susceptor. 

Scanning electron microscopy (SEM, Hitachi SU-70, Hitachi High-Technologies Corporation, Tokyo, Japan) was utilized to analyze the morphology of the CNT forest. Tunneling electron microscopy (TEM, Titan 80-300, FEI Company, Hillsboro, OR, USA) was used to analyze single and multi-walled CNTs (MWCNTs) and Raman spectroscopy (Jobin-Yvon T64000, Horiba Scientific Longjumeau, France) with 515 nm laser was used to characterize the crystallinity of CNTs. 

**Figure 2:**
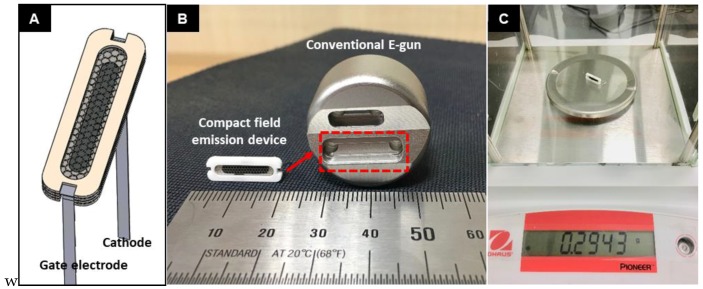
(**A**) 3D design of compact field emission device embedded with directly grown CNT on metal substrate; (**B**) photo image of compact field emission device and conventional dual type electron gun after removing filament emitter; (**C**) photo image of 0.2943 gram-weight of compact field emission device.

The field emission (FE) measurement was done at a base pressure of 10^−7^ Torr in triode condition. Figure 1B describes the set-up of the FE experiment. The Keithley 6485 (Keithley Instruments, Inc., OH, USA) was chosen to measure the cathode current, whereas the Keithley 248 (Keithley Instruments, Inc., OH, USA) was selected for measuring the gate current and as the gate voltage source. The Spellman SL300 (Spellman High Voltage Electronics Corporation, New York, NY, USA) was applied to measure the anode current and as the anode voltage source. Anode voltages of all FE measurements were obtained below 8 kV in pulsed mode with 1 kHz frequency and 50% duty cycle. The turn-on field and threshold field were defined as the gate fields corresponding to current densities of 1 μA/cm^2^ and 1 mA/cm^2^, respectively. For the FE measurement and X-ray image acquisition, the as-grown CNT emitters were assembled into a compact field emission device and then placed into a conventional electron gun as a cathode for the open-type X-ray system, which had been reported in detail elsewhere [12]. Figure 2A illustrates the 3D diagram of the compact field emission device used herein. It consists of a metal mesh that is made up of Kovar and with regular hexagonal sides of 360 µm, an area of 0.00336 cm^2^, and aperture ratio of 89.7%. The CNT emitters inside the compact field emission device were fixed at a distance of 370 μm from the mesh electrode. Figure 2B shows the optical image of the compact field emission device and filament-removed, dual-type conventional electron gun, which also signifies the compactness of the electron gun assembly. The absence of focuser in the compact field emission device has been compensated by the focusing structure (inside the red dashed-line rectangle) of the conventional electron gun. Given that the compact field emission device weighs around 0.3 g (Figure 2C), light-weight is one of its strong attributes. The distance between the emitter and anode was maintained at 2 cm during the FE measurement. For X-ray image acquisition, an integrated circuit board was used. The detector (RAD icon 0889, Teledyne Rad-icon Imaging Corp., CA, USA) with 1024 × 512 pixels was placed 25 cm away from the X-ray system to take high-resolution images. 

## 3. Results and Discussion

### 3.1. CNT Emitter for Compact Field Emission Device

**Figure 3:**
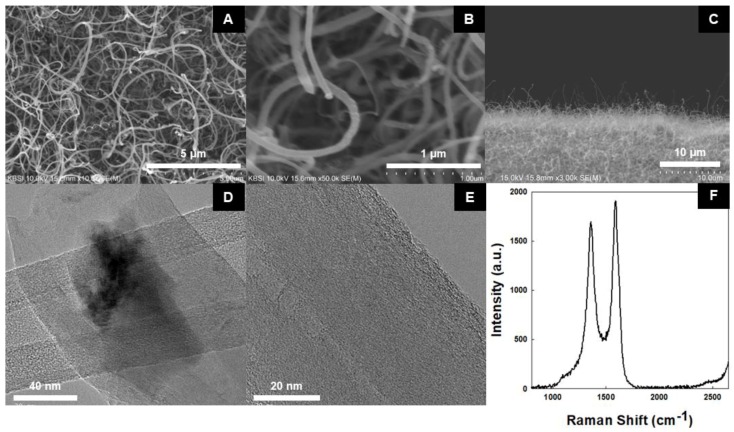
(**A**,**B**) SEM images of CNTs grown on metal substrate synthesized by PECVD; (**C**) lateral SEM Image (**D**) and (**E**) TEM image of CNTs; (**F**) Raman spectrum of CNTs.

Figure 3A,B shows SEM images of CNTs on a metal substrate, taken at 5 and 1 µm scale, respectively. It is evident that the forest of CNTs was densely grown over a large area on the substrate. This is proof that Ni and Fe compounds act as catalysts in the growth of CNTs on the metal alloy substrate. The presence of these catalysts has the exclusion of an extra process required to form a catalyst layer. The CNTs exhibited a spaghetti-like orientation, as shown in Figure 3C. The lack of vertical alignment requires further investigation but appears to be due to the combined effect of substrate roughness and low plasma intensity [23]. Although the dense forest of CNTs was achieved on the metal alloy substrate, the crowding effect was not strong enough to align the CNTs vertically, as is the case for CNTs grown on Si substrates [27]. Since the CNTs were not vertically oriented, it was difficult to measure their exact average length but we have estimated it to be between 20 and 30 µm. Figure 3D shows the TEM image of a CNT tip, where the presence of Ni on the tip signifies tip growth of the CNT. Figure 3E shows that the synthesized multi-walled CNTs (MWCNTs) have a layer thickness of 80 nm with a highly crystalline surface.

### 3.2. Raman Spectrum of PECVD Sythensized CNT

The Raman spectrum of synthesized CNT is shown in Figure 3F. Typically, the first order G band (corresponding to the degree of nanotubes graphitization) and D band (corresponding to the degree of nanotubes structure disorder) for MWCNT are respectively ~1580 and ~1350 cm^−1^ [10,28]. In this Raman spectrum, obtained G and D bands are respectively 1582.8 and 1357.6 cm^−1^, confirming the presence of MWCNTs possessing the intensity ratio ID/IG of 0.81. This is consistent with results obtained for diamond-like carbon (DLC) doped with ZnO [29]. Our results support less disorder in the synthesized CNTs, which is quite anomalous for CNTs synthesized by the PECVD process [30]. Generally, the ID/IG ratio of the PECVD process is greater than 1, but as a result of high temperature (900 °C) synthesis of the CNTs, the degree of disorder decreased significantly as in Chemical Vapor Deposition (CVD) [31]. This preserved the crystallinity of the graphitic surface of the MWCNTs.

### 3.3. Field Emission Measurment Using Compact Field Emission Device

**Figure 4:**
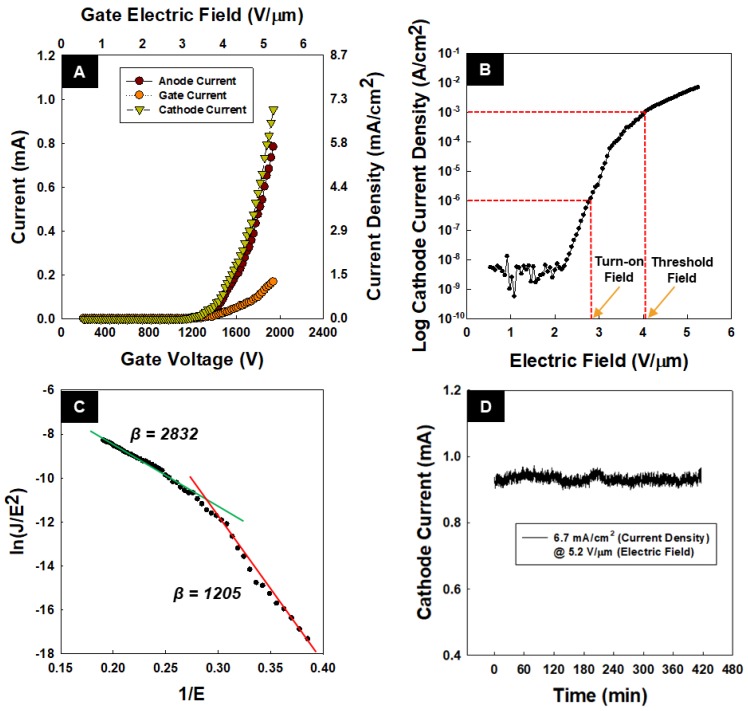
(**A**) I–V curve; (**B**) semi log plot; (**C**) the FN plot; (**D**) stability of CNT emitter.

To measure the FE properties of the CNTs, the electrical aging process was performed for 50 repeatable FE cycles. The electrical aging process was carried out to produce stable FE [32]. Figure 4A illustrates the current density vs. electric field characteristic of the CNT emitters after the electrical aging process. Figure 4B shows the semi-log plot of the cathode current density, showing turn-on (1 μA/cm^2^) and threshold (1 mA/cm^2^) fields of 2.84 and 4.05 V/μm, respectively. The peak value of the current density was 7 mA/cm^2^ at the electric field of 5.24 V/μm for the cathode current. The peak value for the anode and the gate currents were 5.77 and 1.23 mA/cm^2^, respectively. The leakage current of the gate electrode was calculated to be 17.7%. Figure 4C shows the Folwer–Nordheim (FN) plot of the FE of the fabricated CNTs.

To obtain the field enhancement factor β, (i.e., the ratio of the local electric field around the emitter tips and the applied macroscopic electric field) the following equation (FN plot) was used to fit the experimental data [33].

$$\ln\left(\frac{J}{E^{2}}\right)=a-\frac{6.8\times 10^{3}\phi^{3/2}}{\beta E}.$$

Here, J, E, and ϕ, are respectively the cathode current density (mA/cm^2^), electric field between the gate and cathode (V/μm), and the CNT work function (eV) [34]. The work function of the CNTs was assumed to be 4.7 eV [35], whereas their field enhancement factor was calculated to be 2838 at a high electric field (highlighted in the figure by the top solid green line) and 1205 at a low electric field (highlighted in the figure by the bottom solid red line). Figure 4C illustrates the stability of the CNT emitter for 420 min. The initial current density was 6.7 mA/cm^2^ at the electric field of 5.2 V/μm. During 420 min, maximum current density was 7.06 mA/cm^2^, and minimum current density was 6.53 mA/cm^2^. This result indicates high stability of the CNT emitters.

### 3.4. X-ray Image Acqusition Using Compact Field Emission Device Embedded in Conventional Electron Gun 

**Figure 5:**
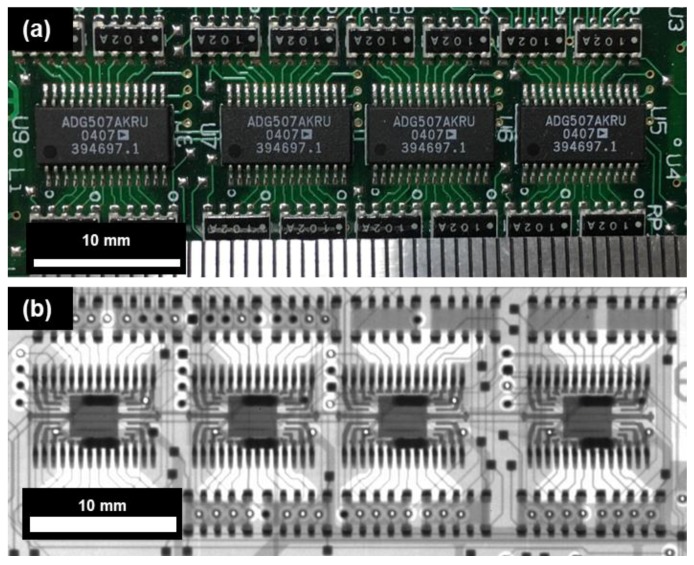
(**a**) The optical image and (**b**) X-ray image of integrated circuit taken at 55 kV/0.5 mA.

Figure 5a,b shows the optical image and X-ray generated image of the integrated circuit by an Open-type X-ray system using CNT field emitters. The X-ray image was taken at an anode accelerating voltage of 55 kV with anode current of 0.5 mA by being exposed for 100 ms. Comparing the scale bars of the optical image and the X-ray image, the high-resolution image under 100 µm was obtained. 

## 4. Conclusions

A cost-effective and simple process to synthesize CNTs on a metal alloy substrate has been demonstrated. Through an optimized PECVD process, a highly stable and high-performance CNT emitter was synthesized. The CNT emitter was custom assembled into a compact and light-weight FE device which is capable of replacing the thermionic emitters in conventional electron guns. The compactly assembled electron gun yielded an enhanced and stable electron FE, which was able to produce a high-resolution X-ray image. Furthermore, this research focuses on improving the FE properties, such as the extraction of more currents at low electric field in the triode configuration and the use of CNT-based electron field emitters for multi X-ray source devices. Applications for the emitter presented herein are limitless, extending to CT scans and tomosynthesis. The target is to replace analog, thermionic filament-based X-ray machines in a sustainable and economic manner.
